# Risk Factors for COVID-19–Related Hospitalization and Death in Patients With Cancer: The National Cancer Institute COVID-19 in Cancer Patients Study (NCCAPS)

**DOI:** 10.1001/jamaoncol.2025.2010

**Published:** 2025-07-17

**Authors:** Brian I. Rini, Ana F. Best, Mel D. Bowman, Grace E. Mishkin, Andrea M. Denicoff, Larry V. Rubinstein, Lyndsay Harris, Ann M. Geiger, Nicholas M. Mark, Steven A. Pergam, Jeremy L. Warner, Alok A. Khorana, Sacha Gnjatic, Tina W.F. Yen, Darla K. Liles, Christine M. Bestvina, Neil J. Shah, Jacqueline T. Norrell, Dawn L. Hershman, Jennifer L. Holter-Chakrabarty, Andrew S. Poklepovic, Stephen J. Chanock, Hari Sankaran, Larissa A. Korde

**Affiliations:** 1 Vanderbilt-Ingram Cancer Center, Nashville, Tennessee; 2 Biometric Research Program, Division of Cancer Treatment and Diagnosis, National Cancer Institute, Bethesda, Maryland; 3 The Emmes Company LLC, Rockville, Maryland; 4 Division of Cancer Treatment and Diagnosis, National Cancer Institute, Bethesda, Maryland; 5 National Cancer Institute Center for Strategic Scientific Initiatives, Rockville, Maryland; 6 Swedish Medical Center, Seattle, Washington; 7 Fred Hutchinson Cancer Center, Seattle, Washington; 8 Warren Alpert Medical School of Brown University, Providence, Rhode Island; 9 Department of Biostatistics, School of Public Health, Brown University, Providence, Rhode Island; 10 Cleveland Clinic, Cleveland, Ohio; 11 Icahn School of Medicine at Mount Sinai, New York, New York; 12 Medical College of Wisconsin, Milwaukee; 13 East Carolina University Brody School of Medicine, Greenville, North Carolina; 14 Hematology/Oncology, Department of Medicine, University of Chicago, Chicago, Illinois; 15 Genitourinary Oncology, Memorial Sloan Kettering Cancer Center, New York, New York; 16 Rutgers Cancer Institute of New Jersey, New Brunswick; 17 Herbert Irving Comprehensive Cancer Center, Columbia University Medical Center, New York, New York; 18 University of Oklahoma College of Medicine, Oklahoma City; 19 Virginia Commonwealth University Massey Cancer Center, Richmond; 20 Division of Cancer Epidemiology and Genetics, National Cancer Institute, Rockville, Maryland

## Abstract

**Importance:**

Retrospective case series have identified having cancer and receiving treatment for cancer as risk factors for inferior COVID-19 outcomes.

**Objective:**

To determine risk factors for hospitalization and death in patients with cancer with COVID-19 infection.

**Design, Setting, and Participants:**

The National Cancer Institute COVID-19 in Cancer Patients Study (NCCAPS) is a prospective longitudinal natural history cohort study examining the impact of COVID-19 on patients with cancer. Adults were eligible within 14 days of an initial positive SARS-CoV-2 test result if they were receiving active treatment for cancer or had prior stem cell/bone marrow transplant or CAR T-cell treatment. The statistical analysis took place between September 2024 and April 2025.

**Main Outcomes and Measures:**

The primary objective of the study was to determine patient factors, therapy types, and cancer types associated with COVID-19 severity, defined as hospitalization for or death from COVID-19 within 30 and 90 days after the first positive SARS-CoV-2 test result. Multivariable regressions were performed for COVID-19–specific hospitalization and mortality (proportional hazard and cause-specific hazard models).

**Results:**

Of 1572 eligible adult patients (median [range] age, 60 [18-93] years; 840 female [53.4%]), 1066 (67.8%) had a solid tumor, with 683 (64.0%) having metastatic disease; breast (252 [23.6%]) and lung cancer (148 [13.9%]) were most common. At enrollment, 1013 patients (64.4%) were unvaccinated for SARS-CoV-2. COVID-19–related mortality at 90 days was 3.0% and did not increase at subsequent time points. The cumulative incidence of COVID-19–specific death in the first 90 days was highest in patients with lymphoma, intermediate in patients with acute leukemia and lung cancer, and lowest in patients with other solid tumors and other hematologic cancers. In multivariable analysis, receipt of chemotherapy (hazard ratio [HR], 1.97; 95% CI, 1.52-2.54) and baseline history of stroke, atrial fibrillation, or pulmonary embolism (HR, 1.78; 95% CI, 1.33-2.38) were associated with a higher risk of hospitalization. Vaccination prior to SARS-CoV-2 infection was associated with a lower risk of hospitalization (HR, 0.52; 95% CI, 0.38-0.70). Over 2 years of follow-up, there were 1739 cancer treatment disruptions, of which 881 (50.7%) were attributed to COVID-19, with most disruptions occurring within the first 30 days.

**Conclusions and Relevance:**

The results of this prospective cohort study showed that COVID-19 had a significant impact on patients with cancer, including hospitalization, treatment disruptions, and death.

## Key Points

**Question:**

What are the risk factors for COVID-19–related hospitalization and death in patients with active cancer receiving treatment and the impact of COVID-19 on patients with cancer?

**Findings:**

In this prospective cohort study of 1572 adults with cancer and a positive SARS-CoV-2 test result, the cumulative incidence of COVID-19–specific death in the first 90 days was highest in patients with lymphoma, intermediate in patients with acute leukemia and lung cancer, and lowest in patients with other solid tumors and other hematologic cancers. In multivariable analysis, receipt of chemotherapy and baseline history of stroke, atrial fibrillation, or pulmonary embolism were associated with a higher risk of hospitalization, and vaccination prior to SARS-CoV-2 infection was associated with a lower risk of hospitalization.

**Meaning:**

COVID-19 infection had a significant impact on patients with cancer, and risk factors for hospitalization and mortality were identified.

## Teaser

This prospective cohort study investigated the impact of COVID-19 on patients with cancer, including risk factors for hospitalization, treatment disruptions, and death.

## Introduction

COVID-19, caused by SARS-CoV-2, has dramatically impacted medical care. The clinical spectrum of COVID-19 ranges from asymptomatic infection to severe illness and death. Older age and comorbidities are associated with more severe presentations and mortality, although severe disease can also occur in those without any risk factors.^1^ Patients with cancer undergoing active therapy are at greater risk for severe SARS-CoV-2 infection and worse outcomes due to immunosuppression from their underlying cancer and/or therapies, as well as age, comorbid conditions, and smoking.^2^

The impact of a cancer diagnosis and/or cancer-directed therapy on COVID-19–related outcomes has been previously reported in retrospective case series. The COVID-19 and Cancer Consortium (CCC19) collected deidentified clinical data on 4966 patients with cancer and polymerase chain reaction (PCR)–confirmed SARS-CoV-2, of whom approximately 80% had solid tumors and 61% had active or recent cancer treatment. Factors associated with COVID-19 severity included demographic factors such as age and sex, comorbidities, type of cancer, and treatment.^3^ The Thoracic Centers International Coronavirus Disease 2019 Collaboration (TERAVOLT) focused on patients with thoracic cancers and reported an all-cause case fatality rate of 24.2%. In that study, performance status, tumor stage, and age were major risk factors.^4^

Patients with cancer also face the possibility that COVID-19 will affect outcomes by disrupting treatment. Dose adjustments and delays are expected when patients with cancer experience infection; however, COVID-19 can result in severe/prolonged illness or protracted viral shedding in individuals with profound immunosuppression, leading to more serious delays or even cessation of cancer treatment, which in turn could impact cancer outcomes.^5^ Thus, long-term data are essential to understanding the effects of the COVID-19 pandemic on patients with cancer.

Prior retrospective studies are limited by the cross-sectional nature of data collection, selection bias, and confounding by indication, potentially limiting generalizability. The National Cancer Institute COVID-19 in Cancer Patients Study (NCCAPS) enrolled patients with cancer and a recent positive COVID-19 test result and followed up prospectively for 2 years to characterize outcomes, describe cancer treatment modifications, and identify patient factors associated with short- and long-term outcomes of COVID-19.

## Methods

This cohort study was reviewed and approved by the NCI Central Institutional Review Board at US sites and by local research ethics boards in Canada. Investigators obtained written informed consent from each participant or each participant’s guardian. The Strengthening the Reporting of Observational Studies in Epidemiology (STROBE) reporting guideline was followed.

### Eligibility

#### COVID-19 Initial Infection

Patient screening and enrollment took place between May 2020 and February 2022. Adult patients with cancer were eligible if they were within 14 days of an initial positive SARS-CoV-2 test result using a nucleic acid PCR test or a rapid antigen test performed using a validated diagnostic assay in accordance with the guidance issued by the US Food and Drug administration (FDA)^6^; serological or antibody tests were not allowed. Any specimen source was allowable.

#### Cancer Therapy

Patients were eligible if they had received cancer treatment within the 6 weeks prior to their first positive SARS-CoV-2 test result, were expected to begin an eligible treatment within 2 weeks, or if they had a prior bone marrow transplant or CAR T-cell therapy. Details of eligible cancer treatment types are included in the eMethods in Supplement 1.

### Data Collection

Patients were recruited from sites participating in 3 NCI clinical trials networks. Longitudinal data collection included information on COVID-19 symptoms, vaccination, treatment, and hospitalization; cancer status and treatment; new comorbidities; and cancer treatment disruption. Race and ethnicity were self-reported. These are routinely collected in NCI-sponsored clinical trials and are particularly pertinent for this analysis because of other studies showing poorer outcomes in Black patients with COVID-19. Clinical data were collected either in-person or remotely at baseline (within 2 weeks of SARS-CoV-2 infection), 2 weeks, 1 month, 2 months, 3 months, 6 months, 9 months, 1 year, and 2 years. Severity of disease was categorized using a 7-point ordinal scale.^7^ Cancer treatments were reported by sites and reviewed and categorized by an author (L.A.K.). For patients who died during the conduct of the study, the cause of death was adjudicated by 2 authors (L.A.K. and B.I.R.) based on the death certificate and medical records as related to COVID-19, related to cancer, other, or unknown. Additional information regarding recruitment, data collection, and adjudication is included in the eMethods in Supplement 1.

### Statistical Analysis

Longitudinal symptom status was assessed as the proportion of patients with a reported symptom category at baseline who also reported a symptom in the same category during a subsequent time point window. Time point windows were defined as 15 to 29 days, 30 to 44 days, 2 months (45-74 days), 3 months (75-104 days), 6 months (135-224 days), 9 months (225-314 days), and 12 months (315-415 days) after the first positive test result. Rates of COVID-19 treatment were tabulated as the proportion of patients per month who received each treatment type within 30 days of their first positive test result. These were plotted using 5-knot binomial splines.

Time-to-event analyses were conducted to evaluate risk factors for severe COVID-19 and death. Cumulative incidence curves were plotted for COVID-19–related vs COVID-19–unrelated death; inpatient admission for COVID-19 treatment; and COVID-19–related death after discharge from hospitalization for COVID-19 treatment, stratified by patient cancer type. Curves for the cumulative incidence of COVID-19–specific deaths among patients hospitalized for COVID-19 treatment were calculated with delayed entry at hospital admission.^8^ Precise definitions of origin, event, censoring, and truncation times are provided in the eMethods in Supplement 1.

Multivariate time-to-event analyses were conducted as proportional cause-specific hazard regressions for hospitalization for COVID-19 and COVID-19–specific death among hospitalized patients with delayed entry at admission. Candidate models evaluated all possible combinations of demographic, baseline medical, and baseline cancer therapy status variables. Models for death among hospitalized patients additionally included baseline COVID-19 therapy status variables. A full description of the procedure and candidate covariates is provided in the eMethods in Supplement 1. Models with the lowest corrected Akaike information criterion value^9^ were selected. Final model *P* values were adjusted using false discovery rate and Bonferroni corrections. Two-sided *P* < .05 was considered statistically significant. Hazard proportionality was evaluated using Schoenfeld residuals. Treatment disruptions were tabulated by reporting time point, and disruptions reported at the 2-week and 1-month time points were further tabulated by type of treatment disruption, reason for disruption, and type of treatment disrupted.

Analyses were conducted using R statistical software, version 4.4.0 (R Project for Statistical Computing), using packages survival, version 3.5-8, and tidyverse, version 2.2.0. The statistical analysis took place between September 2024 and April 2025.

## Results

**Figure 1.**
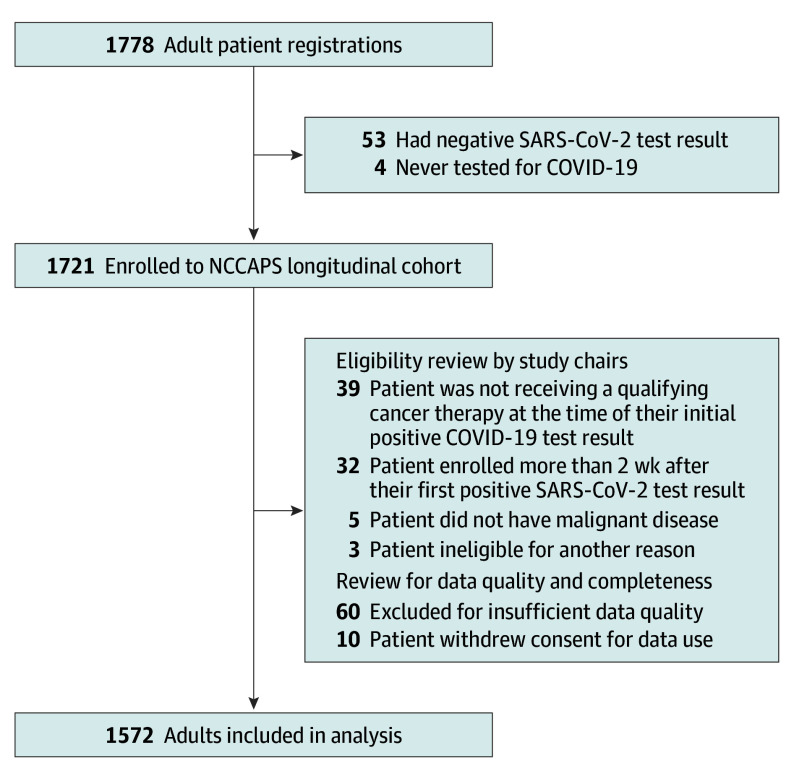
National Cancer Institute COVID-19 in Cancer Patients Study (NCCAPS) Flow Diagram

A total of 2048 patients were screened, and 1778 adult patients enrolled in the longitudinal study; 206 were excluded (136 were ineligible, 10 withdrew consent, and 60 due to insufficient data quality; Figure 1). In total, 1091 (69.4%) provided informed consent remotely. The median (range) age of the 1572 eligible adult patients was 60 (18-93) years, with 587 (37.3%) older than 65 years (Table). Significant baseline comorbidities included 1145 participants (72.8%) with overweight/obesity, 656 (41.7%) with hypertension, 313 (19.9%) with diabetes, and 235 (14.9%) with asthma or chronic obstructive pulmonary disease. The largest population was 683 patients (43.4%) with a metastatic solid tumor, with breast (252 patients [23.6%]) and lung cancer (148 patients [13.9%]) being most common. Chemotherapy was the most common treatment category, with 539 patients (34.3%); 436 patients (27.7%) were receiving targeted therapy; and 167 (10.6%) were receiving immunotherapy. A total of 1013 patients (64.4%) were unvaccinated at the time of their COVID-19 diagnosis, with 472 patients (30.0%) who enrolled prior to vaccine availability. Of those who enrolled after the first SARS-CoV-2 vaccine received emergency use authorization from the FDA, 456 (41.5%) were fully vaccinated, defined as 2 weeks past the second dose of the messenger RNA vaccine (BNT162b2 or mRNA-1273) or a single dose of the adenovirus vector vaccine (Ad26.COV2.S).^10^ Monthly enrollment on the study was consistent with trends in national data reporting patients with COVID-19, including spikes during the emergence of the Delta and Omicron variants of COVID-19 (eFigure 1 in Supplement 1).

**Table.** Baseline Characteristics of National Cancer Institute COVID-19 in Cancer Patients Study (NCCAPS) Participants

Characteristic	No. (%)		
	Total (N = 1572)	Subgroup analysis	
Age, median (range), y	60 (18-93)		
Sex			
Female	840 (53.4)		
Male	732 (46.6)		
Race and ethnicity			
Hispanic	140 (8.9)		
Non-Hispanic Black	173 (11.0)		
Non-Hispanic White	1155 (73.5)		
Other^a^	104 (6.6)		
Body mass index^b^			
<25	427 (27.1)		
25-29	502 (31.9)		
≥30	643 (40.9)		
Smoking status			
Current smoker	123 (7.8)		
Former smoker	615 (39.1)		
Never smoker	809 (51.5)		
Baseline comorbidities			
Hypertension	656 (41.7)		
Asthma/COPD	235 (14.9)		
Diabetes	313 (19.9)		
Cancer type		Metastatic	Nonmetastatic
Solid tumor	1066 (67.8)	683 (64.0)	383 (36.0)
Lung	148 (13.9)	107 (72.3)	41 (27.7)
Breast	252 (23.6)	121 (48.0)	131 (52.0)
Colorectal/anal	106 (9.9)	74 (70.0)	32 (30.0)
Gastric/esophageal	37 (3.5)	21 (56.8)	16 (43.2)
Other GI	59 (5.5)	39 (66.1)	20 (33.9)
Prostate	87 (8.2)	74 (85.0)	13 (15.0)
Other GU	68 (6.4)	57 (83.8)	11 (16.2)
Gynecologic	114 (10.7)	73 (64.0)	41 (36.0)
Skin	33 (3.1)	23 (69.7)	10 (30.3)
Head and neck	23 (2.2)	15 (65.2)	8 (34.8)
Other	139 (13.0)	79 (56.8)	62 (43.2)
Hematologic	506 (32)		
Acute leukemia	99 (19.6)		
Lymphoma	123 (24)		
Multiple myeloma	134 (26.4)		
Other hematologic cancer	150 (29.6)		
Cancer treatment			
Chemotherapy	539 (34.3)		
Targeted therapy (hematologic)	214 (13.6)		
Targeted therapy (solid tumor)	222 (14.1)		
Immunotherapy	167 (10.6)		
Bone marrow transplant/CAR T-cell therapy	105 (6.7)		
Radiation therapy	162 (10.3)		
Endocrine therapy	80 (5.1)		
Other	58 (3.7)		
Vaccination status at enrollment			
Unvaccinated	1013 (64.4)		
Partially vaccinated	67 (4.3)		
Fully vaccinated	456 (29.0)		
Unknown	36 (2.3)		

Abbreviations: CAR, chimeric antigen receptor; COPD, chronic obstructive pulmonary disease; GI, gastrointestinal; GU, genitourinary.

^a^ The other category includes Alaska Native, American Indian, Asian, Native Hawaiian, Pacific Islander, unknown, and not reported.

^b^ Calculated as weight in kilograms divided by height in meters squared.

### COVID-19 Presentation

Of the patients who enrolled in NCCAPS, 1048 (65.1%) were tested for COVID-19 due to symptoms, 259 (15.9%) due to exposure to an individual with known COVID-19, and 197 (8.8%) enrolled after an incidental positive result on a scheduled test. The median (IQR) time from the initial positive SARS-CoV-2 test result to study enrollment was 11 (7-13) days. A total of 1439 patients (91.5%) had at least 1 symptom attributable to COVID-19 within 14 days of their positive SARS-CoV-2 test result. The most common baseline symptoms were respiratory in 229 patients (66.6%) and constitutional in 239 patients (69.5%). Loss of smell or taste at any time point was reported by 358 patients (22.8%). Symptoms generally declined over time, although 184 patients (19.3%) who had symptoms at baseline still had at least 1 symptom at 1 year after enrollment (eFigure 2 in Supplement 1).

### Treatment

**Figure 2.**
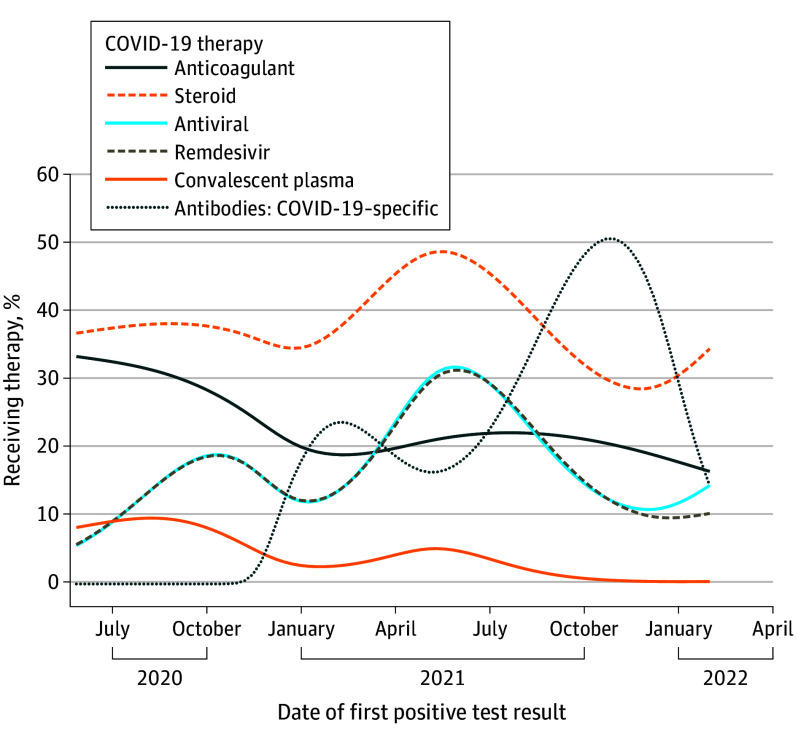
Percentage of Patients Receiving COVID-19 Therapy Within 30 Days of Initial Positive SARS-CoV-2 Test Result Spline methods are described in Supplement 1.

A total of 930 patients (59.2%) received at least 1 treatment for COVID-19, with those who were hospitalized being more likely to receive treatment. A smaller proportion of patients enrolled in 2020 received treatment for COVID-19 than those enrolled in 2021 or 2022 (325 patients [34.9%] vs 317 patients [49.4%], respectively). Use of anticoagulation and convalescent plasma decreased over time, while the use of COVID-19–specific antibodies followed FDA approvals for these agents (Figure 2). Only 31 patients (2%) reported receiving prophylactic COVID-19 antibody infusions (Evusheld) at some point during follow-up. Of note, nirmatrelvir with ritonavir (Paxlovid) received FDA emergency use authorization on December 22, 2021, when the majority of accrual to NCCAPS had already been completed.^11^

### Outcomes

**Figure 3.**
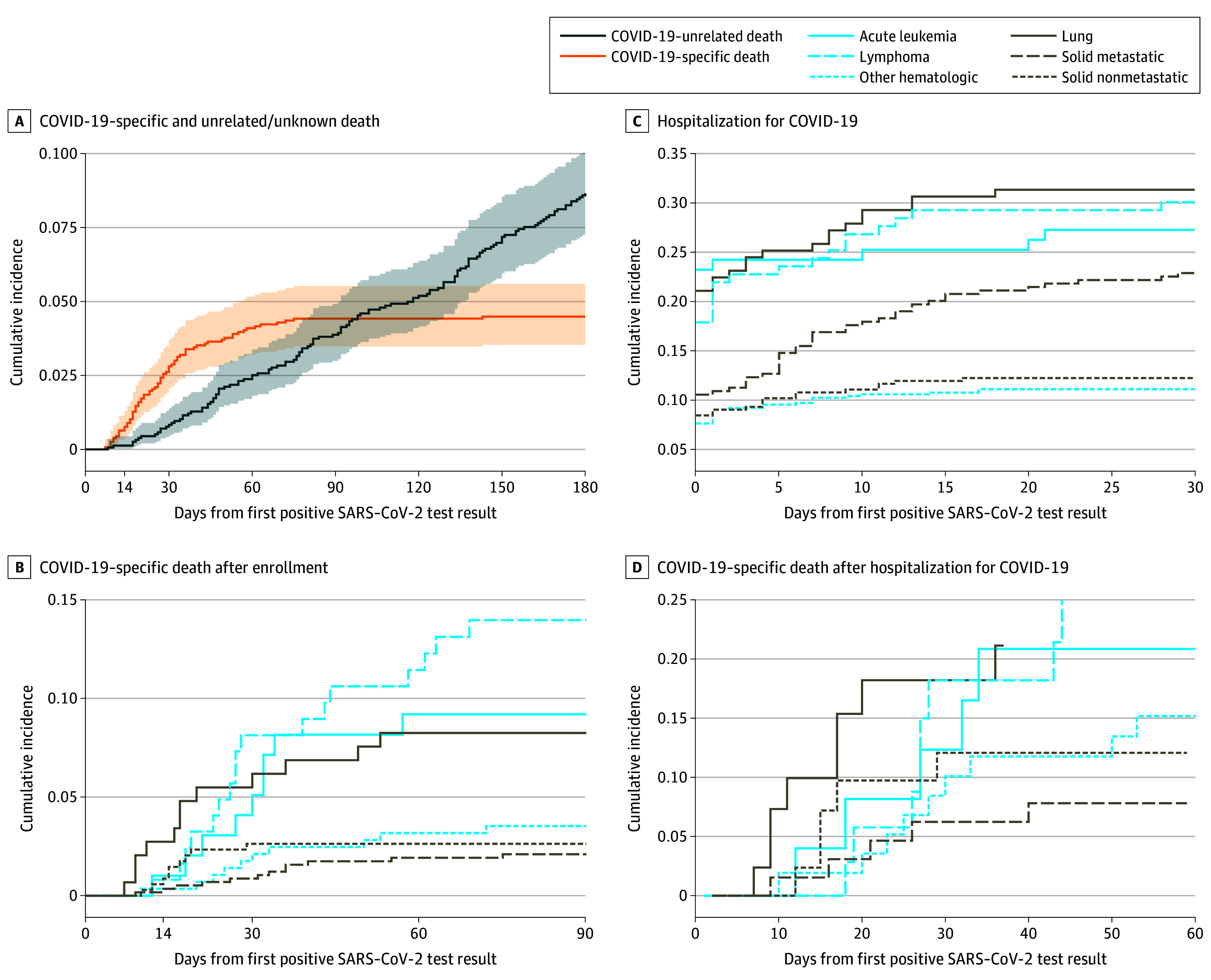
Death and Hospitalization Outcomes After Initial Positive SARS-CoV-2 Test Result The shaded areas in panel A represent 95% CIs. Risk tables for all panels are provided in Supplement 1.

The cumulative incidence of COVID-19–specific and non-COVID-19–related deaths in the first 6 months after enrollment is shown in Figure 3A. COVID-19–related mortality at 90 days was 3.0% and did not increase at subsequent time points; cancer-related mortality was 2.9% at 90 days and increased to 6.6% at 6 months. Given prior data suggesting poorer outcomes among patients with lung cancer, cumulative incidence curves for solid tumors are separated into lung cancer (metastatic and nonmetastatic), other metastatic solid tumors, and other nonmetastatic solid tumors. For hematologic cancers, acute leukemia and lymphoma are presented separately; the other hematologic tumors category includes multiple myeloma, chronic leukemias, and other rare hematologic cancers. The cumulative incidence of COVID-19–specific death in the first 90 days was highest in patients with lymphoma, intermediate in patients with acute leukemia and lung cancer, and lowest in patients with other cancers (Figure 3B).

Overall, 290 patients (18.4%) had at least 1 hospitalization for COVID-19 within 90 days of enrollment. Of the patients who were hospitalized, 68 (23.4%) were admitted to the intensive care unit (ICU). Notably, 18 COVID-19–related deaths (30%) among hospitalized patients were not preceded by ICU admission. Patients with solid non–lung tumors and other hematologic cancers were least likely to require hospitalization for COVID-19 (Figure 3C). Among patients who were hospitalized for COVID-19, the incidence of COVID-19–specific death was highest among patients with lung cancer (Figure 3D). In multivariable regression models, hospitalization for COVID-19 within 30 days was strongly associated with death (hazard ratio [HR], 14.6; 95% CI, 7.6-27.9). Therefore, risk factors for hospitalization and risk factors for death among hospitalized patients were examined separately. Receipt of chemotherapy (HR, 1.97; 95% CI, 1.52-2.54) and baseline history of stroke, atrial fibrillation, or pulmonary embolism (HR, 1.78; 95% CI, 1.33-2.38) were associated with a higher risk of hospitalization. Vaccination prior to SARS-CoV-2 infection was associated with a lower risk of hospitalization (HR, 0.52; 95% CI, 0.38-0.7). Among patients who were hospitalized for COVID-19 within the first 30 days, age was the only factor significantly associated with COVID-19–specific death (for patients 65 years and older: HR, 3.49; 95% CI, 1.58-7.7). The full multivariable analyses are shown in eFigures 3 and 4 and the eTable in Supplement 1.

Thirty-one patients (10.7%) had a second admission for sequelae of COVID-19 within 30 days of their initial admission for COVID-19. In addition, 14 patients who were alive and participating in the study 60 days after their first positive SARS-CoV-2 test result were hospitalized for a subsequent SARS-CoV-2 infection after resolution of their initial COVID-19 presentation.

### Treatment Disruptions

**Figure 4.**
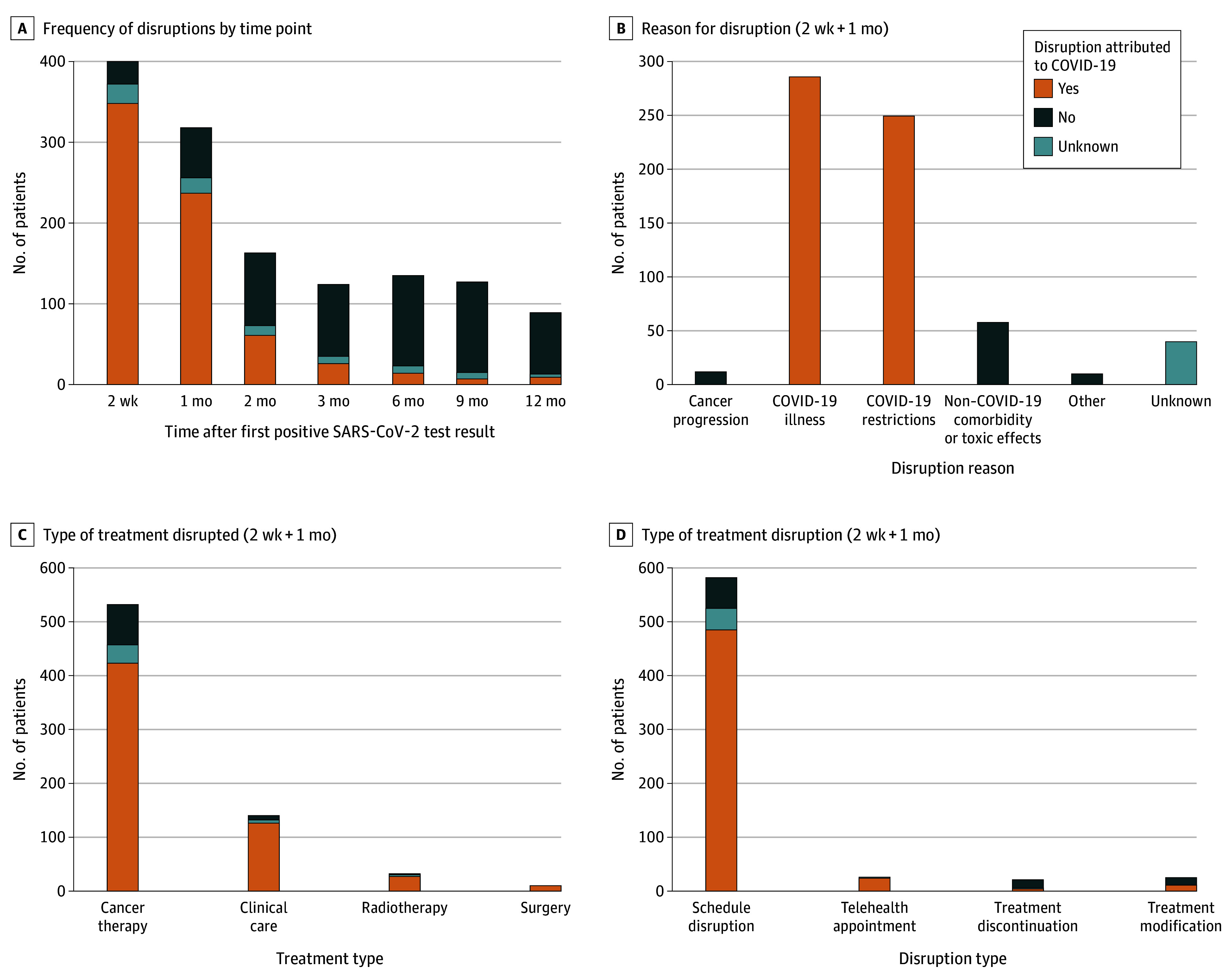
Cancer Treatment Disruption Outcomes Patients with 1 or more treatment disruption(s) were included, stratified by whether the disruption was attributed to COVID-19 or if the attribution status was unknown. The 2-week and/or 1-month posttest time points were used for assessments of reason for disruption (B), type of treatment disrupted (C), and type of treatment disruption (D).

Treatment disruptions were categorized as due to COVID-19 illness (patient too ill due to COVID-19 and/or physician decision to change treatment due to COVID-19), COVID-19 restrictions (institutional restrictions or patient travel restrictions), cancer progression, or non–COVID-19–related. Over 2 years of follow-up, there were 1739 disruptions, of which 881 (50.7%) were attributed to COVID-19, with most disruptions occurring within the first 30 days. Two weeks after the positive COVID-19 test result (2-week posttest time point), 498 patients reported at least 1 treatment disruption, of whom 437 (87.8%) attributed at least 1 disruption to COVID-19. At 1 month after the positive test result, 389 patients reported at least 1 disruption since the 2-week posttest time point, of whom 294 (75.6%) attributed at least 1 disruption to COVID-19. The most common type of disruption was a schedule change (delay) of cancer treatment or clinical care. Figure 4 displays the treatment disruption frequency at various time points, reason for disruption, type of treatment disrupted, and type of disruption.

## Discussion

The COVID-19 pandemic had far-reaching acute and chronic effects on overall health and health care delivery. Patients with cancer were significantly impacted for several reasons. First, as an immunosuppressed population, those with cancer had a high risk of severe disease, as evidenced by the rate of hospitalization. Second, pandemic-related restrictions on health care delivery limited access to cancer treatment and evaluation. Lastly, as lingering symptoms of COVID-19 infection, described as long COVID, have substantial overlap with symptoms of cancer, patients with both cancer and COVID-19 may have experienced more ill effects on quality of life. Although long COVID and quality of life measurements were not formally assessed in this cohort study, both the symptom data and the findings of second hospitalizations for COVID-19 sequelae suggest ongoing effects of the virus beyond the acute phase.

NCCAPS is the only prospective longitudinal assessment of COVID-19 infection in patients with cancer. In this cohort study, patients receiving treatment with chemotherapy and those with a baseline history of thrombotic events such as stroke, atrial fibrillation, or pulmonary embolism had a higher risk of hospitalization for COVID-19. Patients with acute leukemia, lymphoma, and lung cancer were more likely to be hospitalized than those with other cancers. Further, among hospitalized patients, being older than 65 years was associated with death from COVID-19.

The present study confirms findings from other studies in patients with cancer, in which those with lung cancer and hematologic cancers were at the highest risk of poor outcomes, including death.^3,4,13^ As SARS-CoV-2 remains endemic, immunosuppressed populations remain at risk. These data highlight the importance of continued surveillance and prevention strategies in populations at highest risk for complications. Of note, the present study found that patients who were vaccinated had a 50% reduction in risk of hospitalization, even during the early phases of the vaccine rollout. We have previously shown the association between vaccination and COVID-19 disease severity in this population and found that vaccine benefit was present across cancer types.^12^ This is important because there is concern that vaccination response is not as robust in patients with hematologic cancers, particularly those receiving B-cell–depleting therapy.^14,15^ The current data suggest that, despite a potentially suboptimal response, vaccination is still an important preventive strategy in this population, and efforts to ensure patients with cancer had access to vaccines provided important benefits to these patients at high risk for complications from COVID-19.

In NCCAPS, chemotherapy use was associated with a higher risk of hospitalization for COVID-19. Interestingly, the study results did not show an association between outcomes and any treatment type other than chemotherapy. However, the finding that patients with lymphoma have the highest risk of death from COVID-19 suggests a potential detrimental effect of B-cell–depleting therapy on COVID-19 outcomes, although this hypothesis is confounded by the inherent immunosuppression in patients with lymphoma. As the bulk of enrollment occurred prior to the widespread availability of prophylactic antibody treatment, only 2% of the study population received these treatments. It is therefore difficult to ascertain the effect of these interventions on outcomes.

The present data show that, in addition to treatment delays due to acute infection, patients with cancer experienced significant delays in cancer care due to institutional restrictions on care delivery. While it is not possible to estimate the effect of these delays on cancer outcomes with this dataset, others have shown that delays in cancer care affected short- and long-term survival, and that delays in cancer screening and diagnosis during the pandemic led to a stage shift in cancers for which screening is available.^16^ It is yet to be determined whether this transient shift to a more advanced stage at diagnosis will result in higher cancer mortality. Further, a sizable minority of COVID-19–related hospital deaths occurred without ICU transfer. Although the precise reasons for this were not collected, potential causes could be limited ICU bed availability during the most acute phases of the pandemic, pressure to shift goals of care in an advanced cancer population with severe COVID-19 infection, or rapid decompensation before ICU transfer was possible. Notably, these data show that at approximately 90 days, the death rate from cancer exceeded the death rate from COVID-19. Thus, attention to proper delivery of appropriate cancer care is paramount throughout the care continuum, but especially after the acute infection period.

### Limitations

There are several limitations to these data. This study enrolled patients during a period in which several viral variants were prevalent, and detailed information on the specific COVID-19 strain in patients enrolled in the study was not available to provide more granular detail regarding the impact of specific viral strains. Further, although patients in this study were followed up for 2 years, a definitive analysis of the effect of SARS-CoV-2 infection on long-term cancer outcomes would require a comparison of patients with cancer and COVID-19 to those who did not experience infection. As these data were collected through the NCI-sponsored clinical trials networks, which include academic centers and community sites, we believe that the data are broadly representative of patients with cancer in the US and Canada. However, it is possible that patients who enroll in NCI-sponsored clinical trials differ somewhat from the general population of patients with cancer with respect to socioeconomic and/or clinical factors. Therefore, the applicability of these findings to all patients with cancer may be limited by selection bias due to these or other pandemic-related factors. There may be unmeasured confounding arising from patient- and/or site-related factors, precluding strong causal interpretations of the findings. Lastly, as the majority of enrollments occurred prior to FDA approval of nirmatrelvir with ritonavir, the present study does not address whether this treatment affected disease severity.

## Conclusions

The data from this prospective cohort study confirm and expand previous retrospective case series that have found factors, including hematologic cancers, chemotherapy receipt, and lung cancer, as associated with COVID-19 severity. Populations at extreme risk should be carefully followed up as SARS-CoV-2 transitions from a pandemic to an endemic illness.

## Notes

Supplement 1. eMethods eTable. Hazard ratios and corresponding 95% CIs from multivariate models evaluating risk factors for hospitalization for COVID-19 treatment within 30 days of first positive SARS-CoV-2 test result, and death due to COVID-19 after hospitalization within 90 days of first positive test result eFigure 1. Monthly enrollments to NCCAPS compared to new reported COVID-19 cases in the US eFigure 2. COVID-19 symptoms in NCCAPS eFigure 3. Schoenfeld residuals for multivariate Cox proportional hazards model for hospitalization for COVID-19 treatment within 30 days of first positive SARS-CoV-2 test result eFigure 4. Schoenfeld residuals for multivariate Cox Proportional Hazards model for COVID-19–specific death within 90 days after first positive SARS-CoV-2 test result, among patients hospitalized for COVID-19 treatment within 30 days of their first positive test result

Supplement 2. Data Sharing Statement
