# Supplementary material for: Risk Factors for COVID-19–Related Hospitalization and Death in Patients With Cancer: The National Cancer Institute COVID-19 in Cancer Patients Study (NCCAPS)
Source: JAMA Oncol. 2025 Jul 17;11(9):990–8. doi: 10.1001/jamaoncol.2025.2010 (PMC12272355; doi:10.1001/jamaoncol.2025.2010)
Supplement: Supplement 1. — eMethods eTable. Hazard ratios and corresponding 95% CIs from multivariate models evaluating risk factors for hospitalization for COVID-19 treatment within 30 days of first positive SARS-CoV-2 test result, and death due to COVID-19 after hospitalization within 90 days of first positive test result eFigure 1. Monthly enrollments to NCCAPS compared to new reported COVID-19 cases in the US eFigure 2. COVID-19 symptoms in NCCAPS eFigure 3. Schoenfeld residuals for multivariate Cox proportional hazards model for hospitalization for COVID-19 treatment within 30 days of first positive SARS-CoV-2 test result eFigure 4. Schoenfeld residuals for multivariate Cox Proportional Hazards model for COVID-19–specific death within 90 days after first positive SARS-CoV-2 test result, among patients hospitalized for COVID-19 treatment within 30 days of their first positive test result [file jamaoncol-e252010-s001.pdf]

Supplemental Online Content

Rini BI, Best AF, Bowman MD, et al. Risk factors for COVID-19–related hospitalization and death in patients with cancer: the National Cancer Institute COVID-19 in Cancer Patients Study (NCCAPS). *JAMA Oncol.* Published online July 17, 2025. doi:10.1001/jamaoncol.2025.2010

eMethods

eTable. Hazard ratios and corresponding 95% CIs from multivariate models evaluating risk factors for hospitalization for COVID-19 treatment within 30 days of first positive SARS-CoV-2 test result, and death due to COVID-19 after hospitalization within 90 days of first positive test result

eFigure 1. Monthly enrollments to NCCAPS compared to new reported COVID-19 cases in the US

eFigure 2. COVID-19 symptoms in NCCAPS

eFigure 3. Schoenfeld residuals for multivariate Cox proportional hazards model for hospitalization for COVID-19 treatment within 30 days of first positive SARS-CoV-2 test result

eFigure 4. Schoenfeld residuals for multivariate Cox Proportional Hazards model for COVID-19–specific death within 90 days after first positive SARS-CoV-2 test result, among patients hospitalized for COVID-19 treatment within 30 days of their first positive test result

This supplemental material has been provided by the authors to give readers additional information about their work.

© 2025 Rini BI et al. *JAMA Oncology*.

eMethods

Patient Recruitment

Eligible patients were recruited via three NCI-sponsored clinical trials networks: National Clinical Trials Network, consisting of four adult and one childhood cancer cooperative groups in the United States and one adult cooperative group in Canada and represents more than 2200 sites; the NCI Community Oncology Research Program, which includes 7 research bases, 32 community sites, and 14 minority/underserved community sites which together include more than 1,000 affiliate sites across the United States; and the Experimental Therapeutics Clinical Trials Network, comprised of academic centers across the US and Canada. Accrual to this study came from 305 sites in the United States and Canada, as shown below:

Site Name	Screening Accrual Total
Medical College of Wisconsin	69
University of Alabama at Birmingham Cancer Center	51
Memorial Sloan Kettering Cancer Center	37
East Carolina University	35
University of Oklahoma Health Sciences Center	34
University of Chicago Comprehensive Cancer Center	33
Saint Vincent Hospital Cancer Center Green Bay	33
University of Florida Health Science Center - Gainesville	32
Augusta University Medical Center	32
Cooper Hospital University Medical Center	31
Virginia Commonwealth University/Massey Cancer Center	31
Rutgers Cancer Institute of New Jersey	30
NYP/Columbia University Medical Center/Herbert Irving Comprehensive Cancer Center	30
Wayne State University/Karmanos Cancer Institute	29
Vanderbilt University/Ingram Cancer Center	28

© 2025 Rini BI et al. *JAMA Oncology*.

Huntsman Cancer Institute/University of Utah	28
Blank Children's Hospital	24
UPMC Hillman Cancer Center	22
Thomas Jefferson University Hospital	21
Ascension Columbia Saint Mary's Hospital - Milwaukee	21
UT Southwestern/Simmons Cancer Center-Dallas	20
Aspirus Regional Cancer Center	20
ThedaCare Regional Cancer Center	20
University of Kansas Cancer Center	19
Atlanta VA Medical Center	18
Penn State Milton S Hershey Medical Center	18
University of Illinois	16
Indiana University/Melvin and Bren Simon Cancer Center	16
Centro Comprensivo de Cancer de UPR	16
University of Michigan Rogel Cancer Center	15
Nebraska Methodist Hospital	15
Self Regional Healthcare	15
Banner Children's at Desert	14
Veterans Affairs Connecticut Healthcare System-West Haven Campus	14
Oncology Associates at Mercy Medical Center	14
Coborn Cancer Center at Saint Cloud Hospital	14
Summa Health System - Akron Campus	14
Baylor College of Medicine/Dan L Duncan Comprehensive Cancer Center	14
George E Wahlen Department of Veterans Affairs Medical Center	14
University of Wisconsin Carbone Cancer Center - University Hospital	14
Edward Hines Jr VA Hospital	13
CarolinaEast Medical Center	13
Community Medical Center	13
University of New Mexico Cancer Center	13
East Tennessee Childrens Hospital	13
Veterans Affairs Loma Linda Healthcare System	12
CaroMont Regional Medical Center	12
Geisinger Medical Center	12
Spartanburg Medical Center	12
Ben Taub General Hospital	12
UCLA / Jonsson Comprehensive Cancer Center	11
University of California Davis Comprehensive Cancer Center	11
Sarasota Memorial Hospital	11
Lahey Hospital and Medical Center	11
Eastern Maine Medical Center Cancer Care	11
Rice Memorial Hospital	11

© 2025 Rini BI et al. *JAMA Oncology*.

Trinitas Hospital and Comprehensive Cancer Center - Williamson Street Campus	11
Reading Hospital	11
Women and Infants Hospital	11
Saint Vincent Hospital Cancer Center at Saint Mary's	11
Marshfield Medical Center-River Region at Stevens Point	11
CSSS Champlain-Charles Le Moyne	10
UI Health Care Mission Cancer and Blood - Des Moines Clinic	10
Corewell Health Beaumont Troy Hospital	10
Mercy Hospital Springfield	10
University of Texas Health Science Center at San Antonio	10
Augusta Health Center for Cancer and Blood Disorders	10
Zablocki Veterans Administration Medical Center	10
Iowa Methodist Medical Center	9
CoxHealth South Hospital	9
Durham VA Medical Center	9
Ascension Saint Elizabeth Hospital	9
Marshfield Medical Center - Weston	9
Saint Luke's Cancer Institute - Boise	8
Advocate Lutheran General Hospital	8
Saint Jude Midwest Affiliate	8
Ascension Saint Vincent Indianapolis Hospital	8
University of Maryland/Greenebaum Cancer Center	8
Johns Hopkins University/Sidney Kimmel Cancer Center	8
UNC Lineberger Comprehensive Cancer Center	8
Audie L Murphy VA Hospital	8
Covenant Medical Center-Lakeside	8
Valley Medical Center	8
Aspirus Cancer Care - Wisconsin Rapids	8
Kingman Regional Medical Center	7
UC Irvine Health/Chao Family Comprehensive Cancer Center	7
Ingalls Memorial Hospital	7
Sidney and Lois Eskenazi Hospital	7
Goshen Center for Cancer Care	7
University of Kansas Hospital-Westwood Cancer Center	7
Lowell General Hospital	7
MyMichigan Medical Center Midland	7
University of Kansas Cancer Center - North	7
Morristown Medical Center	7
Lankenau Medical Center	7
Methodist Children's Hospital of South Texas	7
Covenant Children's Hospital	7
Adventist Health Cancer Care Center Chico	6
Memorial Health University Medical Center	6

© 2025 Rini BI et al. *JAMA Oncology*.

Lewis Cancer and Research Pavilion at Saint Joseph's/Candler	6
LSU Health Sciences Center at Shreveport	6
Sinai Hospital of Baltimore	6
Eastern Maine Medical Center	6
Henry Ford Hospital	6
Corewell Health Children's	6
University of Minnesota/Masonic Cancer Center	6
Sanford Roger Maris Cancer Center	6
Stony Brook University Medical Center	6
Portland VA Medical Center	6
Geisinger Wyoming Valley/Henry Cancer Center	6
Baptist Memorial Hospital and Cancer Center-Memphis	6
HSHS Sacred Heart Hospital	6
University of Arkansas for Medical Sciences	5
Kaiser Permanente-Vallejo	5
Valley Children's Hospital	5
Gene Upshaw Memorial Tahoe Forest Cancer Center	5
Rocky Mountain Regional VA Medical Center	5
Sibley Memorial Hospital	5
Children's Healthcare of Atlanta - Arthur M Blank Hospital	5
Saint Alphonsus Cancer Care Center-Boise	5
Tufts Medical Center	5
Kaiser Permanente - Largo Medical Center	5
Kaiser Permanente-Woodlawn Medical Center	5
Kaiser Permanente-Gaithersburg Medical Center	5
Dartmouth Hitchcock Medical Center/Dartmouth Cancer Center	5
Alliance for Childhood Diseases/Cure 4 the Kids Foundation	5
Bryn Mawr Hospital	5
Saint Francis Cancer Center	5
Sanford Cancer Center Oncology Clinic	5
Kaiser Permanente-Franklin	4
Colorado Blood Cancer Institute	4
Beebe Health Campus	4
Northeast Georgia Medical Center-Gainesville	4
Saint Luke's Cancer Institute - Twin Falls	4
Loyola University Medical Center	4
Ann and Robert H Lurie Children's Hospital of Chicago	4
University Medical Center New Orleans	4
Henry Ford Health Providence Southfield Hospital	4
Children's Hospital and Medical Center of Omaha	4
Nebraska Cancer Specialists/Oncology Hematology West PC	4
Newark Beth Israel Medical Center	4

© 2025 Rini BI et al. *JAMA Oncology*.

Saint Joseph's Regional Medical Center	4
OptumCare Cancer Care at Charleston	4
James J Peters VA Medical Center	4
ProMedica Toledo Hospital/Russell J Ebeid Children's Hospital	4
Licking Memorial Hospital	4
Oregon Health and Science University	4
WellSpan Health-York Cancer Center	4
University Pediatric Hospital	4
Rhode Island Hospital	4
Parkland Memorial Hospital	4
CHRISTUS Children's	4
Fred Hutchinson Cancer Center	4
Jefferson Healthcare	4
Aurora Saint Luke's Medical Center	4
William S Middleton VA Medical Center	4
Marshfield Medical Center-Marshfield	4
Ascension Columbia Saint Mary's Hospital Ozaukee	4
Aspirus Langlade Hospital	4
Kingston Health Sciences Centre	3
Veterans Administration Medical Center - Birmingham	3
Sharp Memorial Hospital	3
Kaiser Permanente-Fresno	3
UC San Diego Moores Cancer Center	3
Kaiser Permanente-Ontario	3
Phoebe Putney Memorial Hospital	3
Physicians' Clinic of Iowa PC	3
Northwestern University	3
Baystate Medical Center	3
University of Michigan Health - West	3
Park Nicollet Clinic - Saint Louis Park	3
Cardinal Glennon Children's Medical Center	3
University of Kansas Cancer Center at North Kansas City Hospital	3
Wentworth-Douglass Hospital	3
Sidney Kimmel Cancer Center Washington Township	3
Pottstown Hospital	3
AnMed Health Cancer Center	3
South Carolina Cancer Specialists PC	3
Thompson Cancer Survival Center	3
USA Health Strada Patient Care Center	2
Kaiser Permanente-Santa Teresa-San Jose	2
Enloe Medical Center	2
Kaiser Permanente-Santa Rosa	2
Kaiser Permanente-San Diego Zion	2

© 2025 Rini BI et al. *JAMA Oncology*.

Fremont - Rideout Cancer Center	2
Kaiser Permanente-San Marcos	2
UC Irvine Health Cancer Center-Newport	2
Mission Hope Medical Oncology - Santa Maria	2
Medical Oncology Hematology Consultants PA	2
Broward Health Medical Center	2
Saint Mary's Medical Center	2
Moffitt Cancer Center	2
Augusta Oncology Associates PC-D'Antignac	2
Mercy Medical Center - Des Moines	2
Carle Cancer Center	2
Cancer Care Specialists of Illinois - Decatur	2
Richard L. Roudebush Veterans Affairs Medical Center	2
IU Health North Hospital	2
Mercy Hospital Pittsburg	2
University of Kansas Cancer Center-Overland Park	2
Tulane University School of Medicine	2
Mary Bird Perkins Cancer Center - Covington	2
Women's Cancer Care-Covington	2
Mercy Medical Center	2
Corewell Health William Beaumont University Hospital	2
Trinity Health Grand Rapids Hospital	2
McLaren Cancer Institute-Macomb	2
C S Mott Children's Hospital	2
Henry Ford Macomb Hospital-Clinton Township	2
Trinity Health IHA Medical Group Hematology Oncology - Brighton	2
Regions Hospital	2
Abbott-Northwestern Hospital	2
Mercy Hospital	2
Mercy Hospital Saint Louis	2
Saint Louis Cancer and Breast Institute-Ballwin	2
Saint Barnabas Medical Center	2
Rutgers New Jersey Medical School	2
Inspira Medical Center Vineland	2
Ocean University Medical Center	2
Rochester General Hospital	2
Cleveland Clinic Foundation	2
Mercy Hospital Oklahoma City	2
Prisma Health Cancer Institute - Eastside	2
Prisma Health Cancer Institute - Seneca	2
Prisma Health Cancer Institute - Faris	2
BI-LO Charities Children's Cancer Center	2
Gibbs Cancer Center-Pelham	2
UMC Cancer Center / UMC Health System	2

© 2025 Rini BI et al. *JAMA Oncology*.

Driscoll Children's Hospital	2
Kaiser Permanente Tysons Corner Medical Center	2
PeaceHealth Saint Joseph Medical Center	2
Marshfield Medical Center-Rice Lake	2
ThedaCare Regional Medical Center - Neenah	2
Marshfield Medical Center-EC Cancer Center	2
West Virginia University Healthcare	2
Kaiser Permanente-Fontana	1
Kaiser Permanente-Anaheim	1
Kaiser Permanente San Leandro	1
Kaiser Permanente-Walnut Creek	1
Kaiser Permanente Medical Center - Santa Clara	1
Salinas Valley Memorial	1
Kaiser Permanente-Baldwin Park	1
Kaiser Permanente-Stockton	1
Kaiser Permanente-Irvine	1
Kaiser Permanente-Deer Valley Medical Center	1
Kaiser San Rafael-Gallinas	1
Mission Hope Medical Oncology - Arroyo Grande	1
Rocky Mountain Cancer Centers-Littleton	1
Rocky Mountain Cancer Centers-Boulder	1
Kaiser Permanente-Rock Creek	1
Kaiser Permanente-Lone Tree	1
Beebe South Coastal Health Campus	1
Tampa General Hospital	1
Golisano Children's Hospital of Southwest Florida	1
Augusta Oncology Associates PC-Wheeler	1
Saint Alphonsus Cancer Care Center-Nampa	1
Saint Luke's Cancer Institute - Nampa	1
Saint Luke's Cancer Institute - Meridian	1
Springfield Clinic	1
West Suburban Medical Center	1
University of Kansas Clinical Research Center	1
The James Graham Brown Cancer Center at University of Louisville	1
Ochsner LSU Health Monroe Medical Center	1
Our Lady of the Lake Physician Group	1
Louisiana Hematology Oncology Associates LLC	1
Greater Baltimore Medical Center	1
FMH James M Stockman Cancer Institute	1
Maine Medical Partners - South Portland	1
Trinity Health Saint Joseph Mercy Hospital Ann Arbor	1
Corewell Health Farmington Hills Hospital	1
Allegiance Health	1

© 2025 Rini BI et al. *JAMA Oncology*.

Corewell Health Grand Rapids Hospitals - Butterworth Hospital	1
Trinity Health Medical Center - Brighton	1
Cancer and Hematology Centers of Western Michigan - Norton Shores	1
McLaren Cancer Institute-Clarkston	1
Corewell Health Reed City Hospital	1
Trinity Health IHA Medical Group Hematology Oncology Ann Arbor Campus	1
Trinity Health IHA Medical Group Hematology Oncology - Chelsea Hospital	1
Unity Hospital	1
Mayo Clinic	1
Fairview Southdale Hospital	1
Lakeview Hospital	1
Mercy Hospital Joplin	1
University Health Truman Medical Center	1
Overlook Medical Center	1
Jersey City Medical Center	1
Robert Wood Johnson University Hospital Somerset	1
Mount Sinai Hospital	1
Queens Hospital Center	1
Ohio State University Comprehensive Cancer Center	1
Dayton Physician LLC - Englewood	1
Randall Children's Hospital at Legacy Emanuel	1
Fox Chase Cancer Center	1
Doylestown Hospital	1
Jefferson Hospital	1
Asplundh Cancer Pavilion	1
SMC Center for Hematology Oncology Union	1
Shenandoah Oncology PC	1
Providence Sacred Heart Medical Center and Children's Hospital	1
Ascension Mercy Hospital	1
Mayo Clinic Health System-Eau Claire Clinic	1
Ascension All Saints Hospital	1
Saint Agnes Hospital/Agnesian Cancer Center	1
Vince Lombardi Cancer Clinic - Oshkosh	1
Aspirus Medford Hospital	1

Data Collection and Adjudication

Data were collected via electronic case report forms in Medidata RAVE following procedures outlined in the Theradex CTMS Rave User Guide

© 2025 Rini BI et al. *JAMA Oncology*.

(https://www.theradex.com/cmsAdmin/uploads/user-guide-v2-4.pdf). Clinical data were reviewed for consistency and accuracy on an ongoing basis, including review of duplicate fields where possible. Any identified discrepancies were queried, and source documents were requested to adjudicate inconsistencies. Enrolling sites were subject to audit per CTEP standard guidelines.

Eligible Cancer Treatment Types

Cancer treatment eligibility was designed to maximize enrollment of patients who were immunosuppressed due to cancer treatment, and therefore at greatest risk for adverse outcomes with SARS CoV-2 infection.

Allowable cancer treatment types were:

1. For patients with a **hematologic malignancy, CNS malignancy, or metastatic (Stage IV) solid tumor**: chemotherapy, targeted therapy, monoclonal antibodies, immunotherapy, targeted radionuclide therapy, endocrine therapy, or radiation treatment;
2. For patients with a **non-metastatic (Stage I-III) solid tumor**: any systemic treatment or radiation was allowed, with the exception of adjuvant HER2-targeted therapy (trastuzumab, pertuzumab, neratinib, ado-trastuzumab) or endocrine therapy without concurrent chemotherapy;
3. Patients who had received **prior allogenic stem cell/bone marrow transplant or CAR-T cell or other modified cellular therapy** at any time, **autologous stem cell/bone marrow transplant within the past 2 years**, or who were

© 2025 Rini BI et al. *JAMA Oncology*.

receiving **active treatment or prophylaxis for graft vs. host disease** (acute and/or chronic) were eligible.

Cancer Treatment Categorization:

Cancer therapies were reviewed by LK and adjudicated into the following categories: chemotherapy; targeted therapy (hematologic); targeted therapy (solid tumor); anti-PD(L)-1; other immunotherapy; endocrine therapy; radiation; transplant; other.

Ordinal Scale for COVID-19 Severity (from reference #7):

1. Not hospitalized, able to resume normal daily activities

2. Not hospitalized, unable to resume normal daily activities

3. Hospitalized, not on supplemental oxygen

4. Hospitalized, on supplemental oxygen

5. Hospitalized, on high flow oxygen therapy or noninvasive mechanical ventilation

6. Hospitalized, on ECMO or invasive mechanical ventilation

7. Deceased

Hospitalization and Death Adjudication

For patients that died on study, sites were asked whether the death was related to malignancy, COVID-19, or other causes, and were also asked to note whether COVID-19

© 2025 Rini BI et al. *JAMA Oncology*.

was a contributing factor. These data were reviewed by the study physicians (LK and BR). For ambiguous cases, additional clinical data, including death certificates, death summaries (for patients who died while inpatients) and most recent clinical note for those who died outside of the hospital were reviewed. Clinical data from each inpatient hospitalization was also reviewed by LK and BR to categorize the hospitalization into one of the following categories: initial COVID-19, COVID-19 sequelae, possible COVID-19, hospital-acquired COVID-19, not COVID-19 related.

Time-To-Event Definitions

For multivariable proportional hazards models evaluating risk factors for hospitalization for COVID-19 treatment within 30 days of first positive SARS-CoV-2 test, origin time was defined as the day of the patient's first positive SARS-CoV-2 test. Patients were considered to have had an event if they were hospitalized for COVID-19 treatment, and the event time was defined as the day of hospital admission. Patients were censored at loss to follow-up, death prior to hospitalization, or 30 days after first positive test.

For models evaluating risk factors for death among patients hospitalized for COVID-19 treatment within 30 days of first positive SARS-CoV-2 test, origin time was defined as the day of the patient's first positive SARS-CoV-2 test. Models incorporated delayed entry, with left-truncation time defined as the day of hospital admission. Patients were considered to have had an event if they died and their death was attributed to COVID-19, and the event time was defined as the day of death. Patients were censored at loss to follow-up, death from other causes, or 90 days after first positive test.

© 2025 Rini BI et al. *JAMA Oncology*.

For cumulative incidence plots evaluating the risk of hospitalization for COVID-19 sequelae among patients hospitalized for COVID-19 treatment within 30 days of their first positive SARS-CoV-2 test, origin time was defined as the day of discharge from their primary hospitalization. Patients were considered to have had an event if they were hospitalized for COVID-19 sequelae, and their event time was defined as the day of admission for this hospitalization. Patients were censored at loss to follow-up, death, or 90 days after discharge from their primary hospitalization.

For the cumulative incidence plot evaluating the risks of COVID-19-specific vs. COVID-unrelated death, competing risks methods were used to separate these two events. Origin time was defined as the day of first positive SARS-CoV-2 test. Patients were considered to have had an event if they died within 180 days of their first positive SARS-CoV-2 test, and these deaths were separated into competing events by COVID-19 attribution status of the death. Patients were censored at loss to follow-up, or 180 days after their first positive SARS-CoV-2 test.

Model Selection

Model selection was conducted using an exhaustive search of candidate models, evaluated using the corrected form of Akaike's Information Crition (AICc)[9]. Candidate models evaluated all possible combinations of demographic, baseline medical history, cancer therapy. In models for 90-day COVID-specific survival among patients hospitalized for acute COVID-19 treatment, COVID-19 therapy variables were also considered. The following table lists all covariates used in model selection procedures as well as their levels:

© 2025 Rini BI et al. *JAMA Oncology*.

Variable Category	Candidate Covariates
Demographics	Sex (Male, Female)<br><br>Age at Enrollment (18-39, 49-64, 65+) OR (18-64, 65+)<br>Race/Ethnicity (Non-Hispanic White, Non-Hispanic Black, Hispanic, Other, Unknown/Unspecified) OR (Non-Hispanic White, not Non-Hispanic White)<br><br>Time period of first positive test (Early Pre-Vax [5/7/20 – 11/14/20], Late Pre-Vax [11/15/20 – 1/31/21], Alpha Variant [2/1/21 – 7/6/21]. Delta Variant [7/7/21 – 12/24/21], Omicron Variant [12/25/21 – 2/1/22])
Baseline Medical Variables	Malignancy Category (Non-Lung Solid Non-Metastatic, Non-Lung Solid Metastatic, Acute Leukemia, Lymphoma, Lung, Other Hematologic) OR (Non-Metastatic Solid, Metastatic Solid, Hematologic) OR (Non-Hematologic, Hematologic)<br>Baseline BMI Category (Underweight, Normal, Overweight, Obese)<br>History of Asthma and/or COPD (Present, Absent)<br>History of Hypertension (Present, Absent)<br>History of CHF (Present, Absent)<br>History of Diabetes type 1 or 2 (Present, Absent)<br><br>History of Stroke, Afib, and/or PE (Present, Absent)
Cancer Therapy Variables	Chemotherapy within ≤6 wks pre-test or ≤2 wks post-test (Present, Absent)<br>Immunotherapy within ≤6 wks pre-test or ≤2 wks post-test (Present, Absent)<br>Endocrine therapy within ≤6 wks pre-test or ≤2 wks post-test (Present, Absent)<br>Targeted therapy for solid malignancies within ≤6 wks pre-test or ≤2 wks post-test (Present, Absent)<br>Targeted therapy for hematologic malignancies within ≤6 wks pre-test or ≤2 wks post-test (Present, Absent)<br>Radiotherapy within ≤6 wks pre-test or ≤2 wks post-test (Present, Absent)<br><br>Transplant or CAR-T therapy at any time pre-test (Present, Absent)
COVID-19 Therapy Variables	COVID-19-specific antibodies within ≤30d post-test (Present, Absent)<br>Convalescent plasma within ≤30d post-test (Present, Absent)<br><br>Antiviral therapy within ≤30d post-test (Present, Absent)

All potential candidate models (4,718,591 models for 30-day hospitalization, 37,748,728 for 90-day COVID-specific survival among patients hospitalized for acute COVID-19)

© 2025 Rini BI et al. *JAMA Oncology*.

were fit and ordered by the AICc value. In each case, the model with the lowest AICc was selected as the final model and presented here; models within 2 units of this value were evaluated to determine if they had improved clinical interpretability relative to the lowest-AICc model.

COVID-19 Therapy over Time

Due to the non-uniformity of the accrual rate over time, rates of COVID-19 therapy use over the study period were plotted using polynomial splines fitted to the time series of first positive SARS-CoV-2 test dates and treatment status within 30 days of first positive test. Splines were fitted with 5 knots, and visually compared to crude estimates from patients aggregated by month. R function geom_smooth in package ggplot2 was used for these calculations, specifying a binomial glm model and 5 knots.

Risk/Event Tables for Time-To-Event Plots:

The risk/event tables for Figure 3 are provided below:

A) COVID-19-Specific and Unrelated/Unknown Death

Days after First Pos. Test	0	14	30	60	90	120	150	180
Patients at Risk	1572	1556	1506	1441	1404	1364	1328	1291
COVID-19 Deaths	0	12	32	20	5	0	1	0
Unrelated/Unknown Deaths	0	2	11	26	21	20	30	22

B) COVID-19-Specific Death after Enrollment

© 2025 Rini BI et al. *JAMA Oncology*.

Malignancy Category	Days after First Pos. Test	0	14	30	60
Acute Leukemia	Patients at Risk	99	97	93	85
	COVID-19 Deaths	0	1	4	4
Lymphoma	Patients at Risk	123	121	111	106
	COVID-19 Deaths	0	1	9	4
Other Hematologic	Patients at Risk	284	283	277	271
	COVID-19 Deaths	0	1	5	3
Lung	Patients at Risk	147	141	135	123
	COVID-19 Deaths	0	4	5	3
Non-Lung Solid Metastatic	Patients at Risk	576	574	561	530
	COVID-19 Deaths	0	2	3	6
Non-Lung Solid Non-Metastatic	Patients at Risk	343	340	329	326
	COVID-19 Deaths	0	3	6	0

C) Hospitalization for COVID-19

Malignancy Category	Days after First Pos. Test	0	10	20	30
Acute Leukemia	Patients at Risk	99	73	72	70
	COVID-19 Hospitalizations	25	2	1	1

© 2025 Rini BI et al. *JAMA Oncology*.

Lymphoma	Patients at Risk	123	90	87	85
	COVID-19 Hospitalizations	22	11	3	1
Other Hematologic	Patients at Risk	284	233	223	216
	COVID-19 Hospitalizations	31	21	10	5
Lung	Patients at Risk	107	104	99	99
	COVID-19 Hospitalizations	32	12	3	0
Non-Lung Solid Metastatic	Patients at Risk	576	512	505	500
	COVID-19 Hospitalizations	47	17	2	0
Non-Lung Solid Non-Metastatic	Patients at Risk	343	304	298	296
	COVID-19 Hospitalizations	30	9	3	0

D) COVID-19-Specific Death after Hospitalization

This cumulative incidence plot is computed using reweighted observations to account for left-truncation at date of admission. The table provides the effective counts after reweighting. – indicates that the curve is undefined at that time.

Malignancy Category	Days after First Pos. Test	10	20	30	40	50	60
Acute Leukemia	Patients at Risk	26.00000	24.00000	24.00000	21.00000	21.00000	20.00000
	COVID-19 Deaths	0	2	1	2	0	0
Lymphoma	Patients at Risk	33.50364	33.20417	29.67130	31.00694	23.00000	-

© 2025 Rini BI et al. *JAMA Oncology*.

	COVID-19 Deaths	0	2	4	0	2	-
Other Hematologic	Patients at Risk	52.00000	61.00000	61.00000	59.00000	59.00000	57.00000
	COVID-19 Deaths	1	1	4	1	1	1
Lung	Patients at Risk	39.65217	35.20551	34.20551	29.00000	-	-
	COVID-19 Deaths	3	4	0	1	-	-
Non-Lung Solid Metastatic	Patients at Risk	64.43529	64.00000	62.00000	63.33333	62.33333	62.33333
	COVID-19 Deaths	1	1	2	1	0	0
Non-Lung Solid Non-Metastatic	Patients at Risk	40.42424	38.41667	40.83333	40.83333	40.83333	40.83333
	COVID-19 Deaths	0	4	1	0	0	0

© 2025 Rini BI et al. *JAMA Oncology*.

eTable. Hazard ratios and corresponding 95% CIs from multivariate models evaluating risk factors for hospitalization for COVID-19 treatment within 30 days of first positive SARS-CoV-2 test result, and death due to COVID-19 after hospitalization within 90 days of first positive test result

Variable	Level	30-Day Hospitalization		90-Day Post-Hosp. COVID-Specific Death	
		HR	95% CI	HR	95% CI
Basline Vaccination Status	Fully Vaccinated	0.52	(0.38, 0.70)	0.52	(0.2, 1.38)
	Unknown	2.56	(1.56, 4.19)	5.65	(2.07, 15.41)
Sex	Male	1.35	(1.06, 1.73)	1.79	(0.9, 3.57)
Age Category at Enrollment	65+	1.40	(1.10, 1.80)	2.76	(1.41, 5.41)
Race/Ethnicity	Not Non-Hispanic White	1.25	(0.96, 1.62)	-	-
Malignancy Category	Lung	0.96	(0.55, 1.68)	1.16	(0.35, 3.83)
	Lymphoma	1.00	(0.60, 1.66)	2.07	(0.62, 6.87)
	Other Hematologic	0.90	(0.56, 1.44)	1.00	(0.30, 3.41)
	Solid Metastatic	0.46	(0.28, 0.76)	0.47	(0.13, 1.68)
	Solid Non-Metastatic	0.46	(0.27, 0.80)	0.89	(0.25, 3.15)
Baseline History of Asthma/COPD	Present	1.52	(1.14, 2.02)	1.41	(0.68, 2.94)
Baseline History of CHF	Present	1.72	(1.06, 2.77)	1.48	(0.50, 4.39)
Baseline History of Stroke/Afib/PE	Present	1.78	(1.33, 2.38)	1.42	(0.69, 2.92)
Baseline Chemotherapy Status	Current or Recent Tx	1.97	(1.52, 2.54)	1.28	(0.65, 2.53)
Baseline Immunotherapy Status	Current or Recent Tx	1.46	(1.03, 2.08)	-	-
Baseline Heme-Targeted Therapy Status	Current or Recent Tx	1.46	(1.00, 2.15)	-	-

© 2025 Rini BI et al. *JAMA Oncology*.

Baseline Radiotherapy Status	Current or Recent Tx	1.55	(1.08, 2.22)	-	-

- indicates variable was not included in model

© 2025 Rini BI et al. *JAMA Oncology*.

eFigure 1. Monthly enrollments to NCCAPS compared to new reported COVID-19 cases in the US

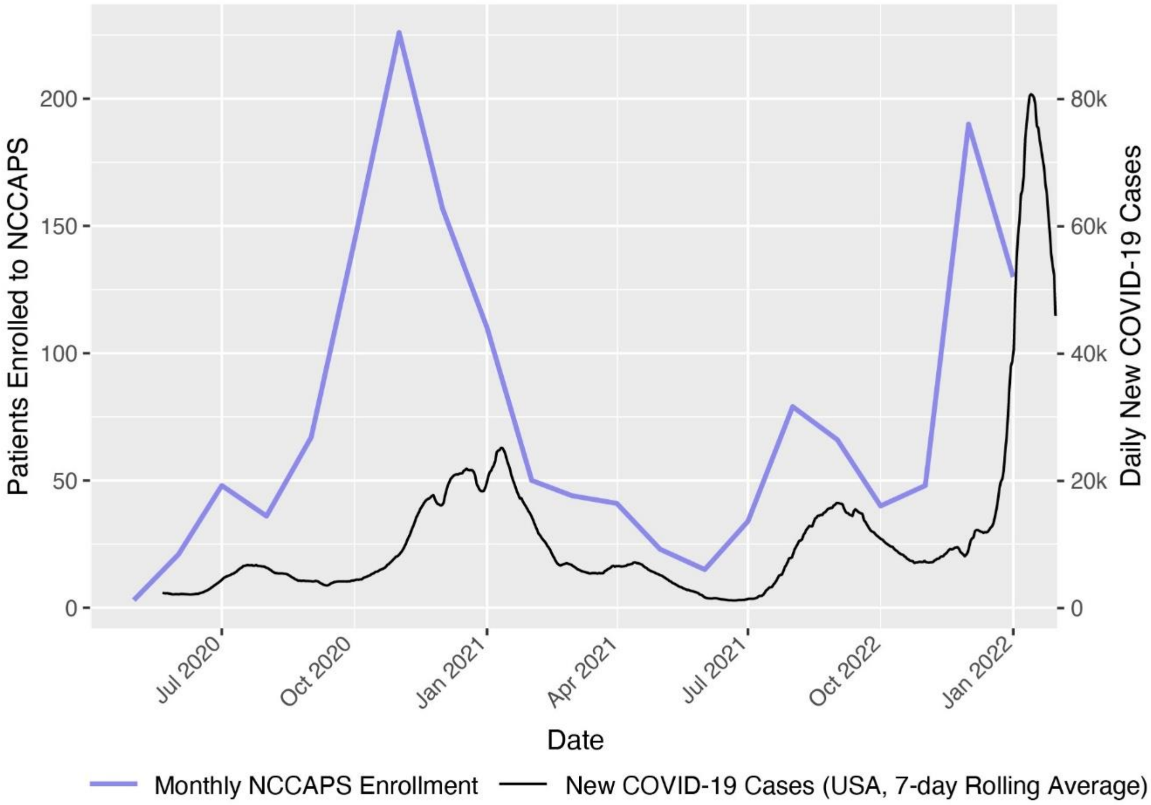

Monthly enrollments to NCCAPS (blue line, left axis) compared to new reported COVID-19 cases in the U.S. (black line, right axis; 7-day rolling average; data from the New York Times).

© 2025 Rini BI et al. *JAMA Oncology*.

eFigure 2. COVID-19 symptoms in NCCAPS

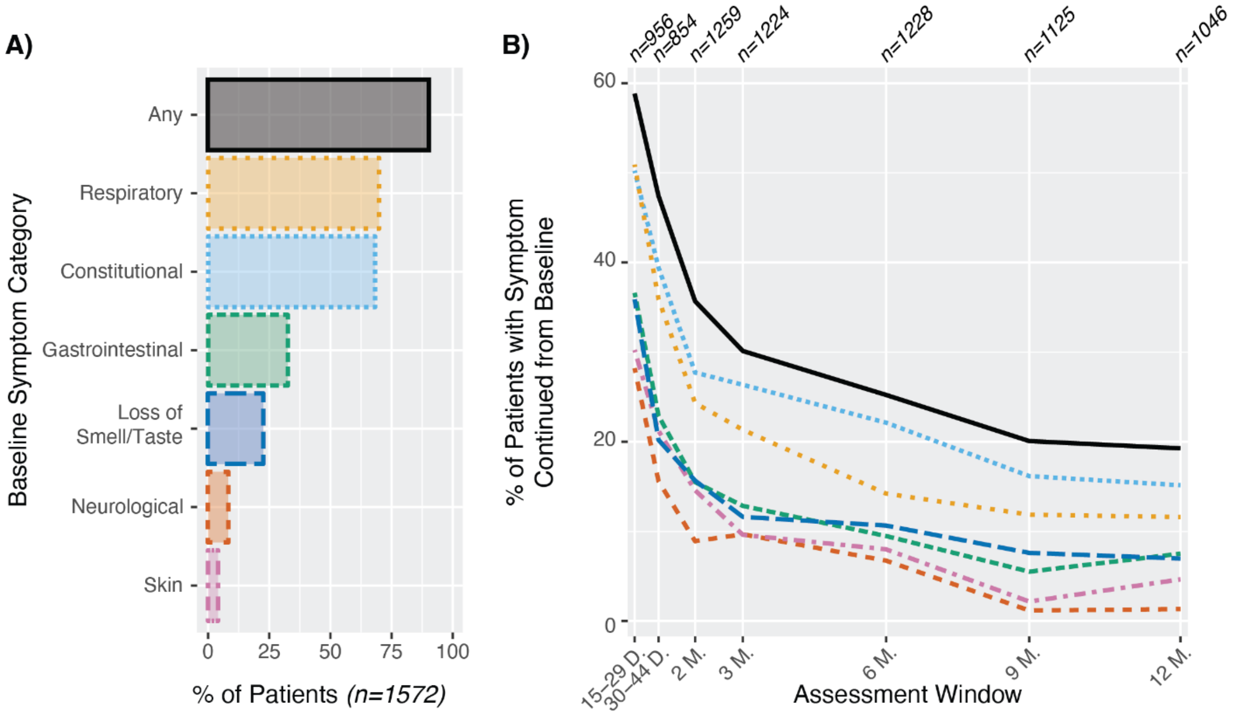

(A) Percent of patients reporting one or more COVID-19 symptoms within 14 days of the first positive SARS-CoV-2 test, by symptom category (B) Percent of patients reporting one or more COVID-19 symptoms who continue to report that symptom during later follow-up, by symptom category and follow-up assessment window

© 2025 Rini BI et al. *JAMA Oncology*.

eFigure 3. Schoenfeld residuals for multivariate Cox proportional hazards model for hospitalization for COVID-19 treatment within 30 days of first positive SARS-CoV-2 test result

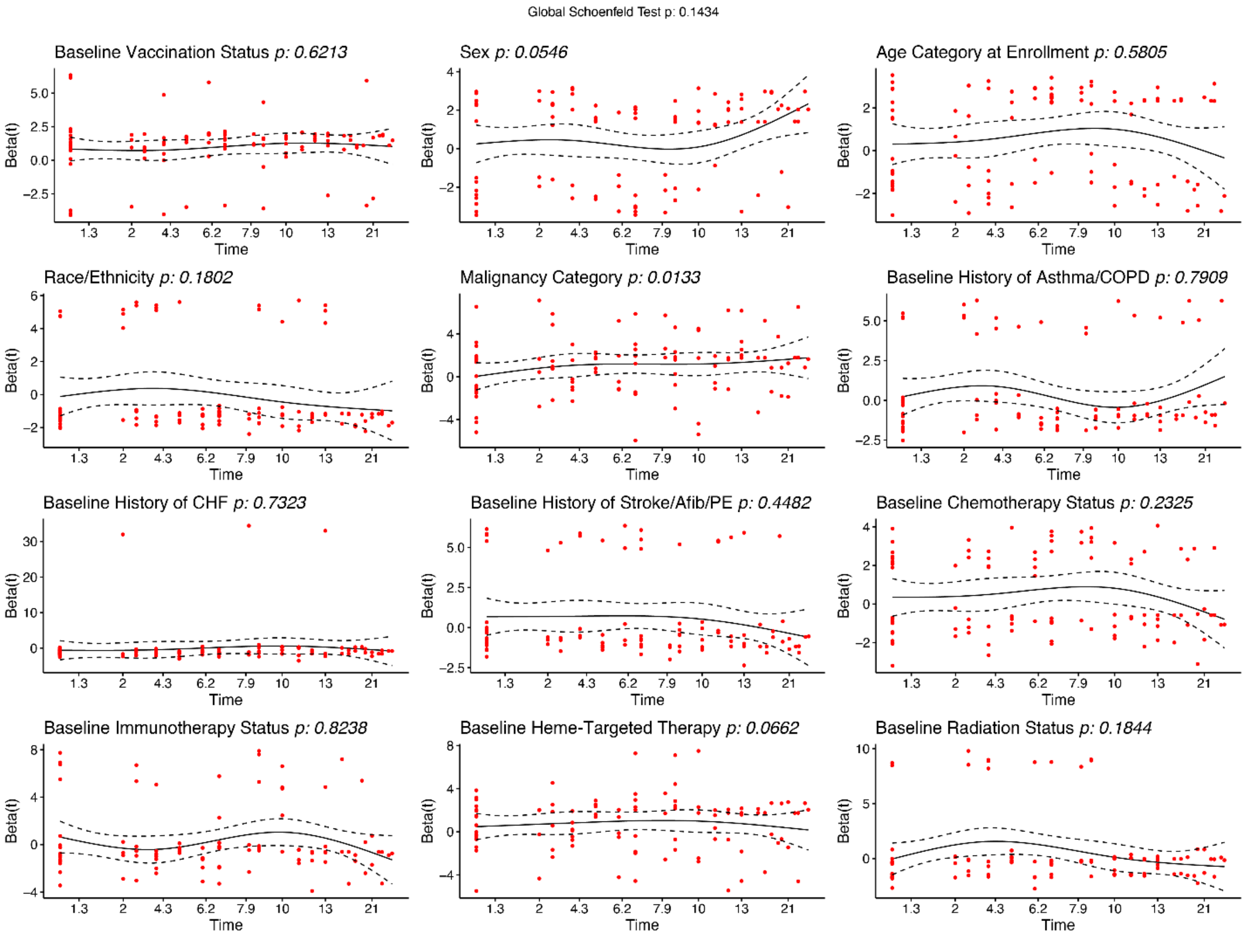

© 2025 Rini BI et al. *JAMA Oncology*.

eFigure 4. Schoenfeld residuals for multivariate Cox Proportional Hazards model for COVID-19–specific death within 90 days after first positive SARS-CoV-2 test result, among patients hospitalized for COVID-19 treatment within 30 days of their first positive test result

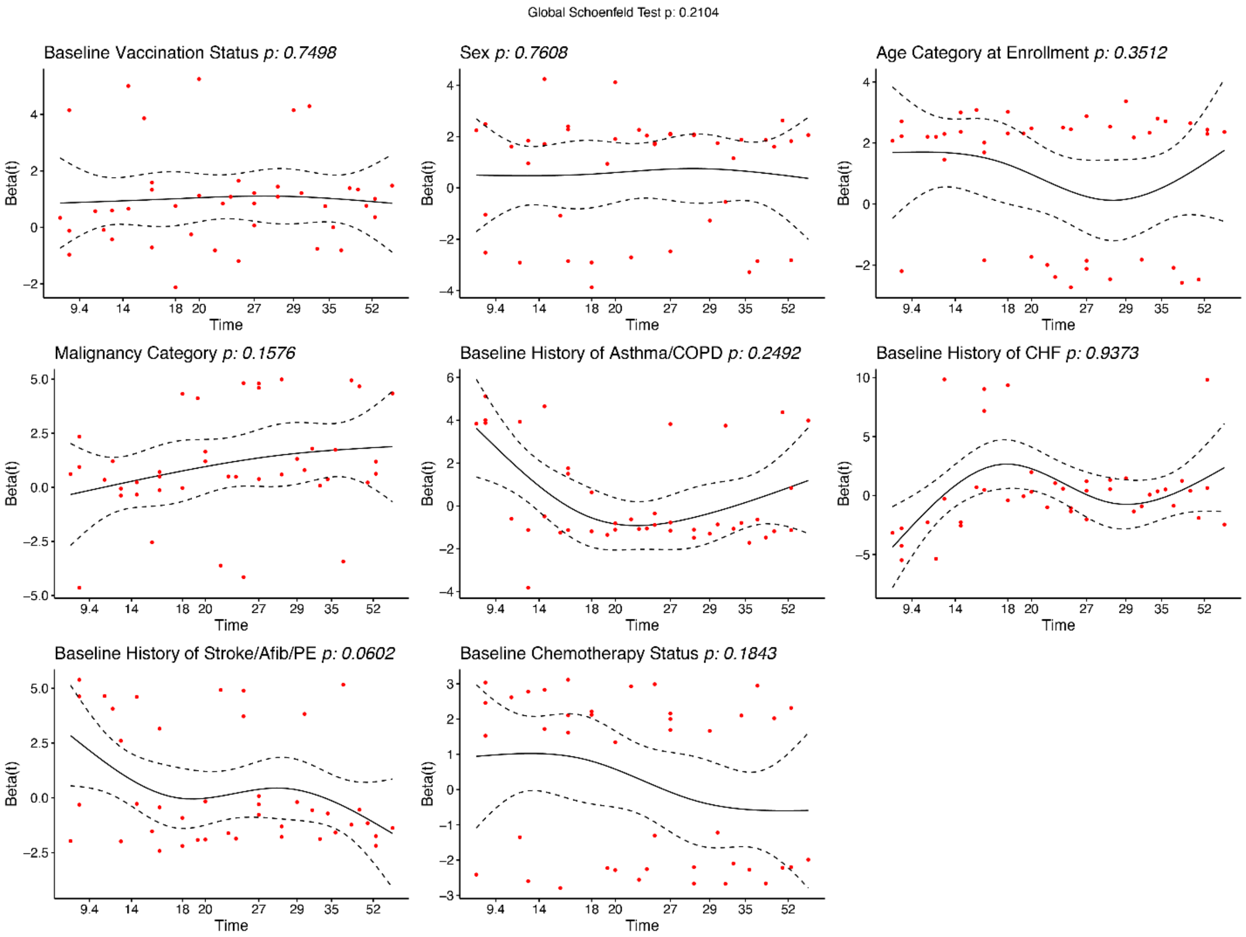

© 2025 Rini BI et al. *JAMA Oncology*.
