# Supplementary material for: Risk Factors for COVID-19–Related Hospitalization and Death in Patients With Cancer: The National Cancer Institute COVID-19 in Cancer Patients Study (NCCAPS)
Source: JAMA Oncol. 2025 Jul 17;11(9):990–8. doi: 10.1001/jamaoncol.2025.2010 (PMC12272355; doi:10.1001/jamaoncol.2025.2010)
Supplement: Supplement 2. — Data Sharing Statement [file jamaoncol-e252010-s002.pdf]

# Data Sharing Statement

Rini. Risk Factors for COVID-19–Related Hospitalization and Death in Patients With Cancer. *JAMA Oncol*. Published July 17, 2025. doi:10.1001/jamaoncol.2025.2010

**Data**
**Data available:** No
